# Assessment of the 1% of Patients with Consistent < 15% Reduction in Low-Density Lipoprotein Cholesterol: Pooled Analysis of 10 Phase 3 ODYSSEY Alirocumab Trials

**DOI:** 10.1007/s10557-018-6784-z

**Published:** 2018-04-07

**Authors:** Harold E. Bays, Robert S. Rosenson, Marie T. Baccara-Dinet, Michael J. Louie, Desmond Thompson, G. Kees Hovingh

**Affiliations:** 1 Departments of Epidemiology & Medicine, Louisville Metabolic and Atherosclerosis Research Center (L-MARC), 3288 Illinois Avenue, Louisville, KY 40213 USA; 2 Cardiometabolics Unit, Mount Sinai Heart, Icahn School of Medicine at Mount Sinai, New York, NY USA; 3 Clinical Development, R&D, Sanofi, Montpellier, France; 4 Regeneron Pharmaceuticals, Inc., Tarrytown, NY USA; 5 Department of Vascular Medicine - Internal Medicine Academic Medical Center, University of Amsterdam, Amsterdam, The Netherlands

**Keywords:** Low density lipoprotein cholesterol, Cholesterol-lowering drugs, PCSK9, Antibodies

## Abstract

**Purpose:**

Clinical trials of statins and other lipid-lowering therapies (LLTs) often report large inter-individual variations in their effects on low-density lipoprotein cholesterol (LDL-C). We evaluated apparent hyporesponsiveness to the proprotein convertase subtilisin/kexin type 9 inhibitor alirocumab (defined as < 15% LDL-C reduction from baseline at all timepoints) using data from 10 Phase 3 trials (3120 hypercholesterolemic patients).

**Methods:**

This report assessed the LDL-C percent reduction from baseline at weeks 4–104 (depending on study), and alirocumab serum levels and antidrug antibodies, in patients with apparent hyporesponsiveness.

**Results:**

Among the 3120 patients evaluated, 98.9% responded to alirocumab, and 33 (1.1%) had < 15% LDL C reduction at all measured timepoints. Pharmacokinetics data indicated that 13/33 apparent hyporesponders had not received alirocumab; no pharmacokinetics data were available for 14/33, and 6/33 had detectable alirocumab. For the six patients with confirmed alirocumab receipt, the degree of adherence to pre-study concurrent LLTs could not be determined after study start; one of these patients had persistent antidrug antibodies.

**Conclusions:**

Apparent hyporesponsiveness to alirocumab appeared to be due to lack of receipt of alirocumab determined by serum alirocumab levels, possible lack of adherence to concurrent LLTs, a theoretical and rare possibility of biological non-responsiveness due to persistent antidrug antibodies, or other causes, as yet unidentified.

**Electronic supplementary material:**

The online version of this article (10.1007/s10557-018-6784-z) contains supplementary material, which is available to authorized users.

## Introduction

Clinical trials of lipid-lowering therapies (LLTs), including statins, often report large inter-individual variations in their effects on low-density lipoprotein cholesterol (LDL-C), including apparent lack of response in some patients. Potential causes of hyporesponsiveness may be lack of receipt of active study drug, changes in concurrent LLTs, inaccurate or unrepresentative baseline lipid levels, or biological non-responsiveness [1]. Race/ethnicity, smoking status, or age may also be factors [2].

Alirocumab is a fully human monoclonal antibody to proprotein convertase subtilisin/kexin type 9 (PCSK9). In high cardiovascular risk patients (including those with heterozygous familial hypercholesterolemia [HeFH]) from Phase 3 ODYSSEY studies, mean LDL-C change from baseline to Week 24 with alirocumab at 150 mg every 2 weeks (Q2W) was − 45.7 to − 61.0% versus + 0.8 to − 6.6% with placebo, and at 75 mg with possible increase to 150 mg Q2W (75/150) at week 12 was − 48% versus + 9.1 to –2.3% [3]. Alirocumab was generally well tolerated in the trials [3, 4]. This analysis of 3120 alirocumab-treated patients with available data from 10 Phase 3 ODYSSEY studies examined apparent hyporesponsiveness to alirocumab, defined as < 15% LDL-C reduction from baseline at all study timepoints wherein blood samples were obtained.

## Methods

This analysis evaluated patients with baseline LDL-C levels ≥ 70 mg/dl pooled from 10 ODYSSEY placebo- or ezetimibe-controlled trials who were treated with alirocumab at 75/150 or 150 mg Q2W, with or without statin and/or other LLTs [5–13]. In the trials, a total of 3120 alirocumab-treated patients were included in the modified intent-to-treat population (all randomized patients with an LDL-C assessment on at least one post-baseline timepoint while on treatment). Trials were at least 24 weeks in duration, with four trials of 78 weeks; one trial was 104 weeks (Supplemental Fig. 1). All trial protocols were approved by the appropriate regulatory body, and all participants were provided written, informed consent.

The LDL-C percent reduction from baseline was examined at weeks 4, 8, 12, 16, 24, 36, 52, 64, 78 (or 76), 88, and 104, depending on the study. The < 15% cutoff for hyporesponsiveness was chosen based on the general minimum LDL-C reduction required for drug approval by the US Food and Drug Administration [14] and was used to define lack of response to PCSK9 inhibitors in previous analyses [15, 16].

Treatment adherence to planned injections of alirocumab and antidrug antibodies were examined in all hyporesponders, as defined above. Serum alirocumab levels (PK_Ali_) were available for a subset of these patients. Alirocumab intake was confirmed when PK_Ali_ was > 10% of the expected alirocumab concentration on at least one timepoint.

### Laboratory Analyses

Patient blood samples for lipid analysis were taken under fasting conditions and were assayed by central laboratories (Medpace Reference Laboratories, Cincinnati, OH, and Leuven, Belgium, except for the LONG TERM study [12], which used Covance Central Laboratories, Indianapolis, IN, and Geneva, Switzerland). Total cholesterol, triglycerides, and high-density lipoprotein cholesterol (HDL-C) levels in serum were determined using Centers for Disease Control and Prevention National Heart Lung Blood Institute Lipid Standardization Program assays. LDL-C was calculated using the Friedewald formula (total cholesterol minus HDL-C minus triglycerides/5); unless triglyceride levels were > 400 mg/dL, in which case it was determined by ultracentrifugation and precipitation (beta-quantification) by the central laboratory. Apolipoprotein B and lipoprotein(a) levels in serum were determined using immunonephelometry. Alirocumab and PCSK9 concentrations in serum were quantified with validated enzyme-linked immunosorbent assays (Regeneron Pharmaceuticals Inc., Tarrytown, NY, USA) [17]. Serum samples were tested for anti-drug antibodies to alirocumab using a validated immunoassay; samples positive for anti-drug antibodies were screened for neutralizing antibodies using a validated immunoassay (Regeneron Pharmaceuticals Inc., Tarrytown, NY, USA) [18].

## Results

**Fig. 1:**
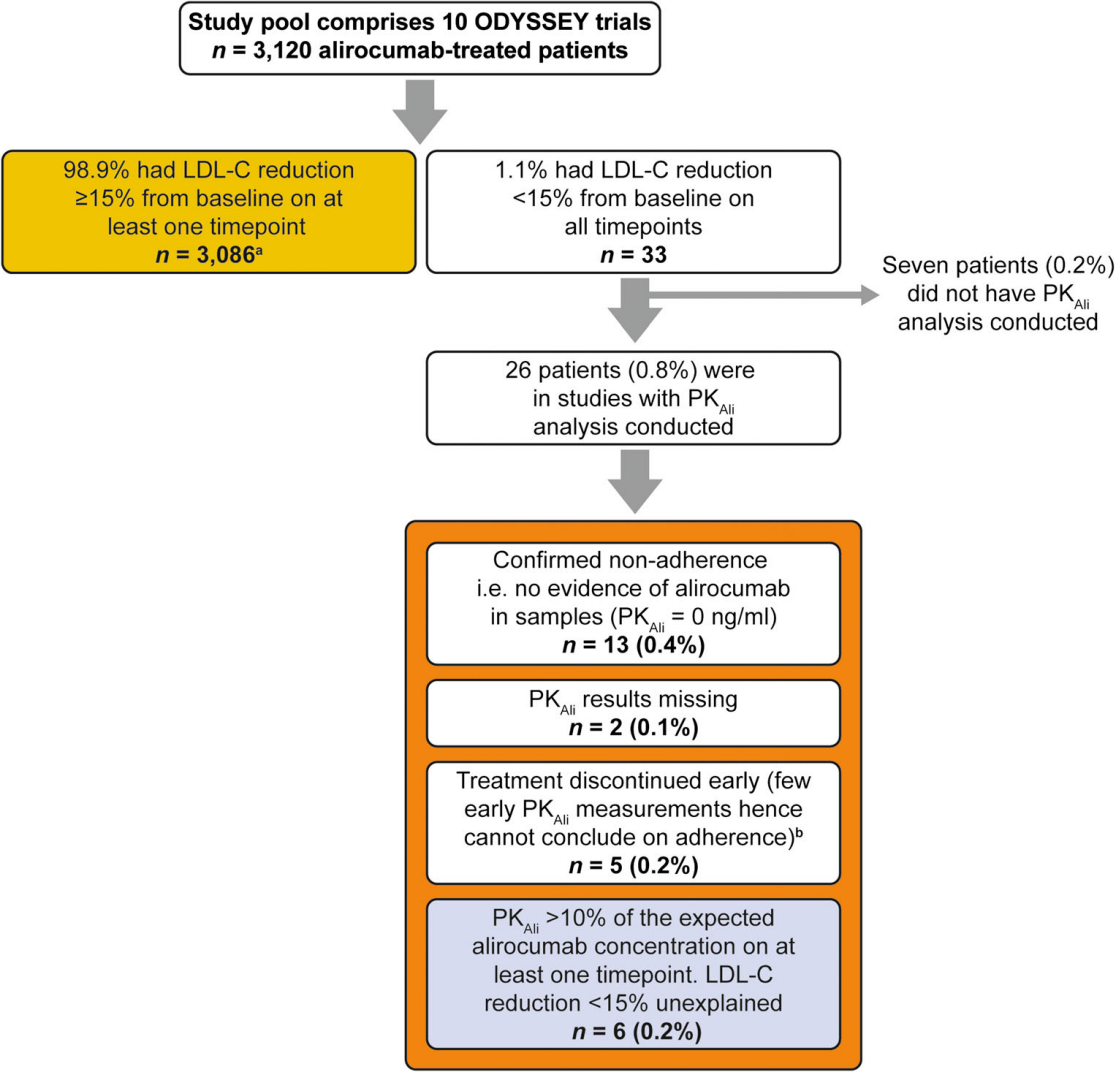
Flow chart of LDL-C response to alirocumab treatment pooled from 10 Phase 3 ODYSSEY trials. ^a^Excluding one patient without post-baseline LDL-C data. ^b^PK_Ali_ data available for only one or two timepoints (Weeks 0, 4, and/or 8) as patient discontinued treatment early. Due to early treatment discontinuation, no conclusions can be made based on the PK_Ali_ findings in those patients. Pooled on-treatment data from 10 Phase 3 ODYSSEY trials: COMBO I, NCT01644175; LONG TERM, NCT01507831; HIGH FH, NCT01617655; FH I, NCT01623115; FH II, NCT01709500; COMBO II, NCT01644188; MONO, NCT01644474; ALTERNATIVE, NCT01709513; OPTIONS I, NCT01730040; and OPTIONS II, NCT01730053. *LDL-C*, low-density lipoprotein cholesterol; *PK*_*Ali*_, serum alirocumab levels

Alirocumab at 75/150 mg Q2W resulted in LDL-C reduction of ≥ 15% in 91% of the patients at week 12 (before dosage increase) and in 92% of the patients at week 24 (Supplemental Fig. 2A). Approximately 95% of the patients treated with alirocumab at 150 mg Q2W observed an LDL-C reduction of ≥ 15% at weeks 12 and 24 (Supplemental Fig. 2B). Across all studies, alirocumab produced an LDL-C reduction of < 15% on at least one timepoint in ~ 25% of patients (Supplemental Fig. 3); however, only 1.1% (*n* = 33) had < 15% LDL-C reduction at all timepoints. Patient diary/caregiver reports indicated ≥ 80% adherence to planned alirocumab injections in all 33 patients; seven of these 33 patients were from studies with no pharmacokinetics analysis per protocol. Of the 26 patients where PK_Ali_ data were available, 13 had undetectable PK_Ali_, suggesting no intake of alirocumab. Two had PK_Ali_ values missing, and five discontinued treatment early (Fig. 1).

The remaining six patients with < 15% LDL-C reduction had detectable PK_Ali_ levels. All were White, with ages ranging 20–63 years; five had HeFH [15] (Table 1). Per protocol, all six patients were to have continued concurrent maximally tolerated statin after study entry; however, adherence to concurrent statins was not specifically monitored. This presented a potential confounder in that if high-intensity statin was discontinued after the study had started, then it would be expected to contribute to subsequent intra-individual LDL-C increases, potentially negating the apparent efficacy of alirocumab. Overall adherence to alirocumab was reported as > 80% in all six patients, but only two completed treatment up to the end of the trial. One patient who reported being treated with atorvastatin 80 mg per day at study start had persistent antidrug antibodies but stopped treatment at week 36 due to poor adherence to protocol (Table 1). It is unknown whether the patient also had poor adherence to concurrent statin. Persistent antidrug antibodies were found in < 2% of alirocumab-treated patients in the 10 Phase 3 trials, which did not seem to affect efficacy in the broad patient population [18].

**Table 1:** Description of the six patients with pharmacokinetics levels compatible with administration of alirocumab but unexplained lack of LDL-C response

Patient^a^	1	2	3	4	5	6
Trial	HIGH FH	HIGH FH	COMBO II	FH I	FH I	LONG TERM
Gender	Female	Female	Male	Female	Female	Female
Age, years	20	34	63	50	42	52
Race	White	White	White	White	White	White
Ethnicity	Not Hispanic or Latino	Not Hispanic or Latino	Hispanic or Latino	Not Hispanic or Latino	Not Hispanic or Latino	Not Hispanic or Latino
Diagnosis of HeFH^b^	Yes	Yes	Non-FH	Yes	Yes	Yes
Mutation	*LDLR* negative c.1-?_67 + ?del	*LDLR* defective p.Val429Met	Not sequenced	*LDLR* defective Pro685Leu	*LDLR* defective p.Asp227Glu	No mutation found
Responders with same mutation	Yes	Yes	N/A	Yes	Yes	N/A
Diabetes	No	Insulin resistance	Type 2	No	No	No
Statin (at randomization)	ROS 20 mg	SIM 40 mg	ATV 80 mg	ROS 20 mg	SIM 40 mg	ATV 10 mg
MTD^c^	Yes	No (due to regional practice/local investigator)	Yes	Yes	No (muscle symptoms and or ↑ CK)	No (due to regional practice/local investigator)
Number of alirocumab administrations^d^	38	8	16	7	20	39
Treatment adherence (%)^d^	95.3	100	84.6	96.2	100	98.5
Persistent ADAs^e^	No	No	Yes	No	No	No
Timepoint of last alirocumab administration	Week 76 (up to the end of the study)	Week 14	Week 36	Week 12	Week 38	Week 76 (up to the end of the study)
Main reason for stopping treatment (if discontinued early)	Not applicable	Adverse event (infections and infestations)	Poor adherence to protocol	Subject withdrew consent	Poor adherence to protocol	Not applicable
Baseline lipid parameters						
LDL-C (calculated)	194 mg/dl (5.02 mmol/l)	216 mg/dl (5.59 mmol/l)	73 mg/dl (1.89 mmol/l)	280 mg/dl (7.25 mmol/l)	181 mg/dl (4.69 mmol/l)	121 mg/dl (3.12 mmol/l)
Apo B	133 mg/dl	120 mg/dl	75 mg/dl	102 mg/dl	106 mg/dl	81 mg/dl
HDL-C	69 mg/dl (1.79 mmol/l)	42 mg/dl (1.09 mmol/l)	49 mg/dl (1.27 mmol/l)	86 mg/dl (2.23 mmol/l)	79 mg/dl (2.05 mmol/l)	55 mg/dl (1.43 mmol/l)
Triglycerides	74 mg/dl (0.84 mmol/l)	79 mg/dl (0.89 mmol/l)	90 mg/dl (1.02 mmol/l)	53 mg/dl (0.60 mmol/l)	79 mg/dl (0.89 mmol/l)	81 mg/dl (0.92 mmol/l)
Lp(a)	5 mg/dl	44 mg/dl	12 mg/dl	114 mg/dl	12 mg/dl	69 mg/dl
Baseline free PCSK9^f^	Not available	Not available	210 ng/ml	250 ng/ml	258 ng/ml	236 ng/ml

*Apo*, apolipoprotein; *ADA*, antidrug antibody; *ATV*, atorvastatin; *CK*, creatine kinase; *HDL-C*, high-density lipoprotein cholesterol; *HeFH*, heterozygous familial hypercholesterolemia; *LDL-C*, low-density lipoprotein cholesterol; *LDLR*, low density lipoprotein receptor; *Lp*(*a*), lipoprotein (a); *MTD*, maximally tolerated statin dose; *non-FH*, non-familial hypercholesterolemia; *PCSK9*, proprotein convertase subtilisin/kexin type 9; *ROS*, rosuvastatin; *SIM*, simvastatin

^a^Arbitrary patient number assigned

^b^Clinical and genotyping criteria [15]

^c^Atorvastatin 40–80 mg, rosuvastatin 20–40 mg, or simvastatin 80 mg, unless there was an investigator-approved reason for using lower doses

^d^Based on patient diary/caregiver reports (except patient 1 where alirocumab administrations were at study site). Overall adherence was calculated for each patient as 100 − (% days with below-planned dosing + % days with above-planned dosing). Below-planned dosing was defined as the number of days with no injection administered within the previous 17 days divided by the duration of treatment-injection exposure in days. Above-planned dosing defined as the number of days with > 1 injection administered within the previous 11 days divided by the duration of treatment-injection exposure in days

^e^≥ 2 consecutive positive samples for ADAs over ≥ 12 weeks

^f^PCSK9 data only from COMBO II, FH II, LONG TERM, and HIGH FH

## Discussion

Among the 3120 patients evaluated, 98.9% had ≥ 15% LDL-C lowering (responsiveness) to alirocumab. Of the 33 patients with apparent hyporesponsiveness, 27 had undetectable or missing alirocumab levels, absence of pharmacokinetics analyses, or early treatment discontinuation. Whether these patients had received alirocumab could not be concluded, either because no pharmacokinetics analysis was performed or there were too few PK_Ali_ values.

### Limitations

This post-hoc analysis, as well as the studies included in the analysis, was not designed to assess adherence to concurrent LLTs such as statins or ezetimibe. Alirocumab pharmacokinetics data were not planned per protocol in all studies.

### Clinical Implications

Non-responsiveness to fully human PCSK9 monoclonal antibodies is rare. When non-responsiveness to PCSK9 monoclonal antibodies does occur, a worry among clinicians is the possible presence of anti-drug antibodies, especially given that PCSK9 monoclonal antibodies are biologics with antigenic potential. A prior report by Shapiro et al. [19] evaluated potential causes of hyporesponsiveness among 17 adults with cardiovascular disease (*n* = 14) and/or familial hypercholesterolemia (*n* = 9) treated with a PCSK9 inhibitor (12 patients received alirocumab and 5 patients received evolocumab). The authors concluded that because total PCSK9 levels typically rise with inhibition of PCSK9 via monoclonal antibodies (due to the antibody binding to PCSK9 in the circulation), this may assist in diagnosing potential causes of hyporesponsiveness. Such an approach may be advantageous in that PCSK9 levels are commercially available through specialty laboratories accessible to clinicians. Conversely, PCSK9 monoclonal antibody levels, and levels of anti-drug antibodies to PCSK9 monoclonal antibodies, are typically available only within the research setting. Furthermore, the presence of anti-drug antibodies does not necessarily mean the anti-drug antibody diminishes the effectiveness of the PCSK9 monoclonal antibody treatment. In fact, because “neutralizing” anti-drug antibodies are often defined by how antibody binding takes place, and not defined by their clinical effects, then even the presence of “neutralizing” anti-drug antibodies to PCSK9 inhibitor monoclonal antibodies may not necessarily account for diminished lipid-lowering effects. Finally, this current report suggests that in the event anti-drug antibodies to PCSK9 inhibitors do occur, their presence is, at best, a very rare potential cause of hyporesponsiveness. Other potential causes are far more likely.

All things considered, how might clinicians best evaluate a patient with apparent hyporesponsiveness to a PCSK9 monoclonal antibody? Based upon this data, one practical approach may be for the patient and medical staff to begin with a 4-week off PCSK9 inhibitor “stabilization” period, wherein other potential lipid-altering confounders are stabilized, including optimal management of diabetes mellitus, adherence to thyroid replacement therapy, and no significant change in nutrition or physical activity. During this 4-week period while off PCSK9 monoclonal antibody therapy, the medical staff should ensure an accurate 4-week pill count of concurrently administered statin and/or other lipid-altering drugs. This can be facilitated by patient self-records of pill intake, family-witnessed/documented lipid-altering drug intake, pill bottle counts by the medical staff, and pharmacy reconciliation of lipid-altering drugs and doses. Also during this 4-week stabilization, the medical staff should directly obtain, and properly store, the prescription PCSK9 monoclonal antibody. Once it is ensured that the patient was stabilized for 4 weeks, that the patient has adhered to concurrent lipid-altering drug therapies for 4 weeks, and that functional PCSK9 monoclonal antibody is ready for use, then a baseline lipid profile blood level [including LDL-C and lipoprotein(a)] should be obtained directly by the medical staff (not an outside laboratory). The patient should then receive PCSK9 monoclonal antibody administered by another highly trained medical staff member—verified and witnessed by an independent highly trained medical staff member. The same lipid profile should then be repeated at 2 and 4 weeks afterwards, while maintaining optimal management of secondary causes of dyslipidemia, maintaining no significant change in nutrition or physical activity, and a strict pill count to absolutely ensure the patient is maintaining concurrently administered statin and/or other lipid-altering drugs. Based upon the current report above, such a practical approach is likely to resolve the vast majority of cases of apparent hyporesponsiveness to a PCSK9 monoclonal antibody.

If, despite the above approach, the reduction in LDL-C levels remains < 15%, then based upon the findings of Shapiro et al., PCSK9 levels could be obtained within 1–5 days after administration of PCSK9 monoclonal antibody therapy, and repeated at least 2 weeks off PCSK9 monoclonal antibody therapy. If the plasma PCSK9 on:off ratio is < 2 (or PCSK9 < 1200 ng/ml), then this suggests a lack of systemic exposure to the PCSK9 monoclonal antibody, as might occur from inactive drug, poor training of and poor injection technique by the patient and medical staff, dermatologic pathology impairing systemic absorption, problems with drug target recognition, or possible anti-drug antibodies. Conversely, if the plasma PCSK9 on:off ratio is > 2 or PCSK9 > 1200 ng/ml, then this suggests the patient has received the intended PCSK9 monoclonal antibody therapy. Potential causes of LDL-C hyporesponsiveness to functional PCSK9 monoclonal antibody therapy in patients with systemic exposure include potential exaggerated PCSK9 secretion, or mutations/dysfunction of PCSK9, LDL receptor, apolipoprotein B, and/or apolipoprotein E.

## Conclusion

In this analysis, treatment hyporesponsiveness in lowering LDL-C could be attributed to lack of receipt of alirocumab, possible lack of adherence to concurrent LLTs, a theoretical, rare possibility of biological non-responsiveness due to persistent antidrug antibodies, or other causes, as yet unidentified.

## Electronic supplementary material

ESM 1 (DOCX 3704 kb)
