# Supplementary material for: Assessment of the 1% of Patients with Consistent < 15% Reduction in Low-Density Lipoprotein Cholesterol: Pooled Analysis of 10 Phase 3 ODYSSEY Alirocumab Trials
Source: Cardiovasc Drugs Ther. 2018 Apr 7;32(2):175–80. doi: 10.1007/s10557-018-6784-z (PMC5958153; doi:10.1007/s10557-018-6784-z)
Supplement: Supplementary file 1 — (DOCX 3704 kb) [file 10557_2018_6784_MOESM1_ESM.docx]

Supplemental Figure 1. Study design of the 10 ODYSSEY Phase 3 Trials


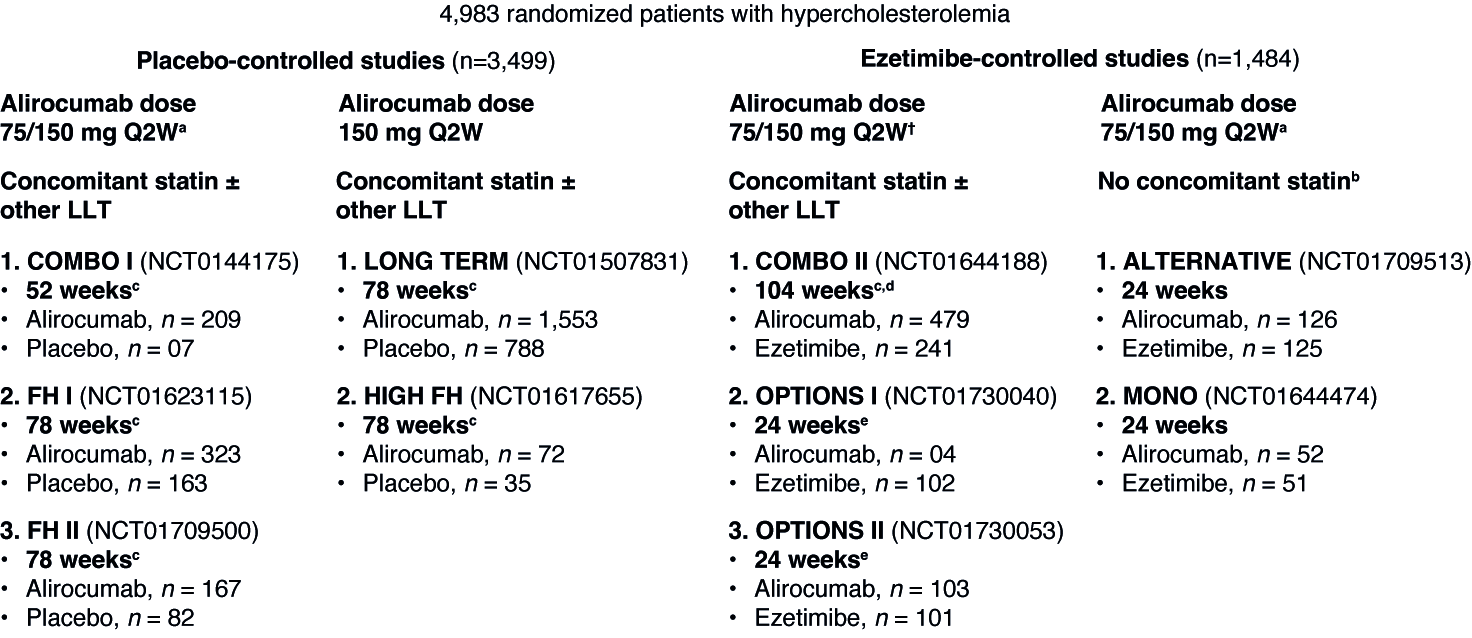


^a^75/150 mg Q2W indicates starting dose of 75 mg Q2W increasing to 150 mg Q2W at Week 12, if pre-specified LDL-C goal was not met at Week 8.

^b^Concomitant non-statin LLT (excluding ezetimibe) allowed in ALTERNATIVE; no concomitant LLT allowed in MONO.

^c^Concomitant statin at maximally tolerated doses (defined as atorvastatin 40–80 mg, rosuvastatin 20–40 mg, or simvastatin 80 mg; lower doses were allowed with an investigator-approved reason).

^d^No concomitant non-statin LLT allowed in COMBO II.

^e^Concomitant statin and doses were atorvastatin 20 or 40 mg in OPTIONS I, and rosuvastatin 10 or 20 mg in OPTIONS II. Concomitant treatment with other statins, ezetimibe, fibrates (other than fenofibrate), and red yeast rice products was prohibited.

LDL-C, low-density lipoprotein cholesterol; LLT, lipid-lowering therapy; Q2W, every 2 weeks.

Supplemental Figure 2. Waterfall plot of percentage change in calculated LDL-C from baseline up to Week 12 and Week 24 with alirocumab at A) 75/150 mg Q2W and B) 150 mg Q2W doses


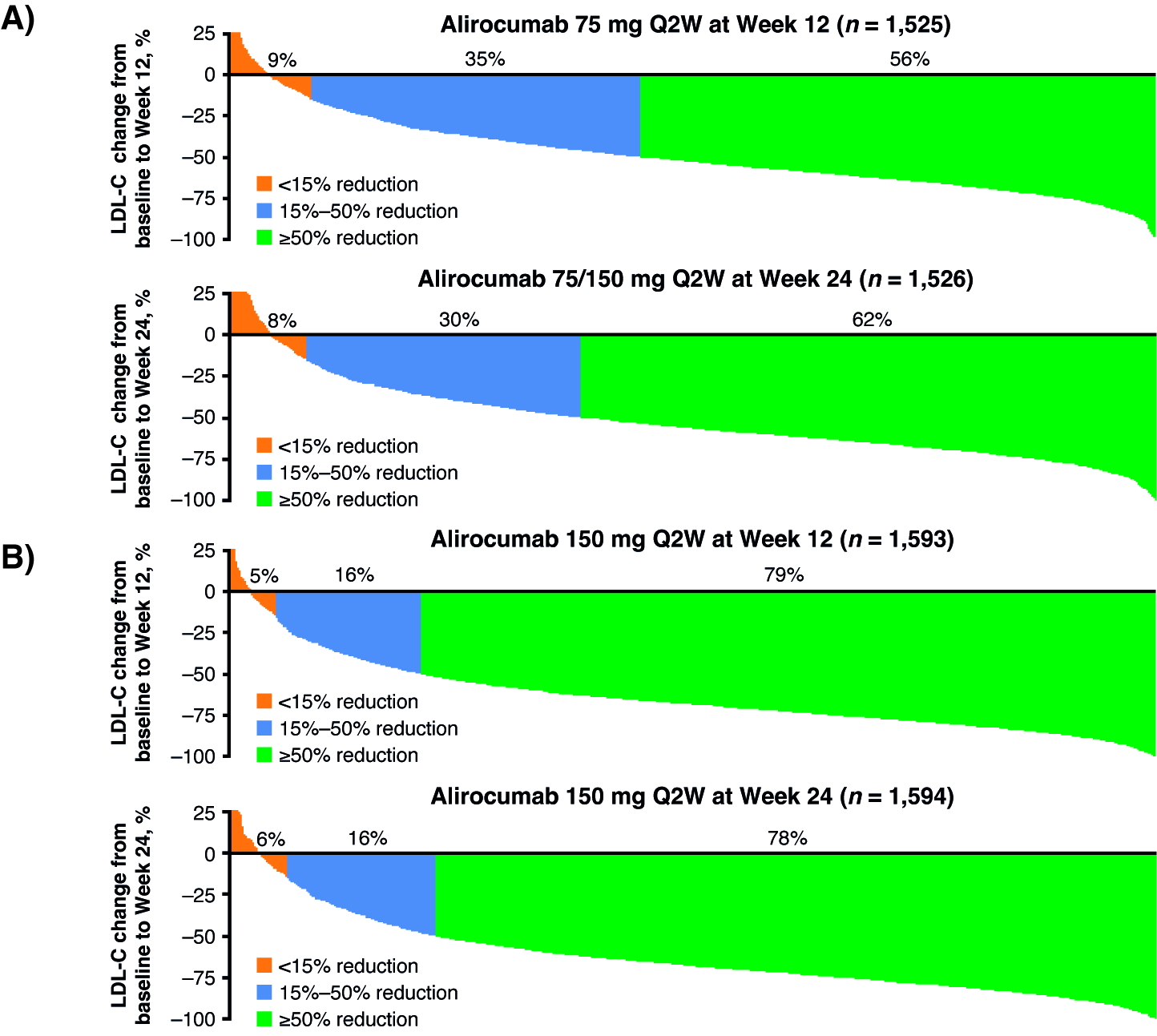


Waterfall plots based on calculated LDL-C values in the modified-intent-to-treat population. Missing data at Week 24 imputed using last value on treatment.

LDL-C, low-density lipoprotein cholesterol; Q2W, every 2 weeks.

Supplemental Figure 3. Pooled analysis of the percentage of alirocumab-treated patients with <15% LDL-C reduction for varying timepoints


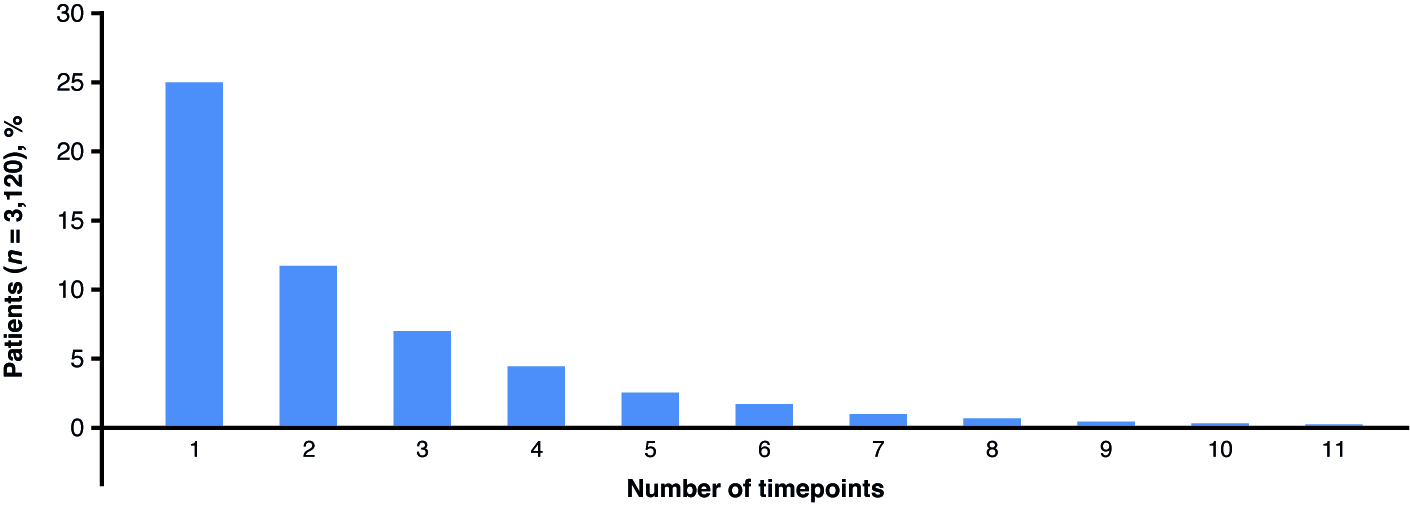


Data analyzed in the modified-intention-to treat population.

LDL-C, low-density lipoprotein cholesterol.
